# Scaling Proprioceptor Gene Transcription by Retrograde NT3 Signaling

**DOI:** 10.1371/journal.pone.0045551

**Published:** 2012-09-19

**Authors:** Jun Lee, Andreas Friese, Monika Mielich, Markus Sigrist, Silvia Arber

**Affiliations:** 1 Biozentrum, Department of Cell Biology, University of Basel, Basel, Switzerland; 2 Friedrich Miescher Institute for Biomedical Research, Basel, Switzerland; Hertie Institute for Clinical Brain Research - University of Tuebingen, Germany

## Abstract

Cell-type specific intrinsic programs instruct neuronal subpopulations before target-derived factors influence later neuronal maturation. Retrograde neurotrophin signaling controls neuronal survival and maturation of dorsal root ganglion (DRG) sensory neurons, but how these potent signaling pathways intersect with transcriptional programs established at earlier developmental stages remains poorly understood. Here we determine the consequences of genetic alternation of NT3 signaling on genome-wide transcription programs in proprioceptors, an important sensory neuron subpopulation involved in motor reflex behavior. We find that the expression of many proprioceptor-enriched genes is dramatically altered by genetic NT3 elimination, independent of survival-related activities. Combinatorial analysis of gene expression profiles with proprioceptors isolated from mice expressing surplus muscular NT3 identifies an anticorrelated gene set with transcriptional levels scaled in opposite directions. Voluntary running experiments in adult mice further demonstrate the maintenance of transcriptional adjustability of genes expressed by DRG neurons, pointing to life-long gene expression plasticity in sensory neurons.

## Introduction

The assembly of neuronal circuits represents a sequential process during which neuronal subpopulations are first specified by cell-intrinsic programs at early developmental stages [1], [2], [3], [4], [5], before neuronal differentiation is further influenced by target-derived signals [6], [7]. Intrinsic cell type specific differences are reflected at the gene expression level, and neuronal subtypes express unique genes controlling initial axon guidance decisions. Little is known about how profoundly target-derived signals influence these initial transcriptional profiles at the genome-wide level in neuronal subpopulations.

Dorsal root ganglia (DRG) sensory neurons innervate various peripheral end organs and relay functionally distinct information to the CNS [8], [9]. Cell-type specific differences at the gene expression level include transcription factors and transmembrane receptors [1], with neurotrophic factor receptors and their ligands as one of the best-understood signaling systems [10], [11], [12]. Functionally distinct DRG populations express the tyrosine kinase receptors TrkA and TrkC, and the target-derived factors nerve growth factor (NGF) and Neurotrophin-3 (NT3) regulate differential neuronal survival [10], [11], [12], [13], [14]. Coincident mutation of neurotrophins and the proapoptotic gene *Bax* allowed elucidation of survival-independent roles of Neurotrophin signaling pathways [14], [15], [16], revealing important roles for NGF signaling in peripheral target invasion [15] and for NT3 signaling in the establishment of central proprioceptor projections [16]. The expression of the ETS transcription factor *Etv1* in proprioceptors is regulated by NT3 [16], [17], identifying one downstream regulator in this cascade. Since proprioceptors are a minority of DRG neurons [18], [19], it remains unknown how they respond more generally to retrograde NT3 signaling by adjusting gene expression [14], and whether variation in NT3 level can modulate transcription specifically within proprioceptors.

Here we exploit a genetic tool to purify and compare the genome-wide proprioceptor transcriptome from different *NT3* signaling mutant mouse strains. By analyzing genes with proprioceptor-enriched expression, we find that elimination of *NT3* signaling in *NT3^−/−^Bax^−/−^* mice [16], [20] profoundly dampens expression of many of these genes. To identify genes acting as possible sensors detecting peripheral NT3 levels, we analyzed proprioceptor gene expression in mice with surplus peripheral NT3 [21], . Anticorrelative analysis identifies a subset of proprioceptor-enriched genes with opposing transcriptional regulation, and uncovers a member of the GABAA receptor gene family *Gabrg1* as a gene with exquisite sensitivity to NT3 signaling. Our study identifies genome-wide transcription profiles enriched and regulated by NT3 signaling in proprioceptors, and provides evidence for pronounced roles of target-derived factors in the regulation of neuronal subtype-specific transcriptional programs.

## Materials and Methods

### Mouse Genetics

Transgenic mice analyzed in this study were *TrkC^GFP^* [23], *Bax^−/−^* [24], *NT3^−/−^* [20], *Mlc^NT3^* [21], and *TrkC-/-* [25]. *Mlc^NT3^* mice exhibit increased numbers of proprioceptive neurons in lumbar DRG, without coincident increase in the frequency of small diameter neurons [21]. Wild-type mice and intercrosses were maintained on a mixed genetic background (129/C57Bl6). All animal experiments were carried out in accordance with the Swiss Guidelines for animal experimentation and approved by the Kantonal Veterinaeramt, Basel, Switzerland.

### DRG Neuron Dissociation and Isolation by Fluorescent Activated Cell Sorting

DRG were exposed through dissection by ventral laminectomy and isolated in ice cold Hank’s balanced salt solution (HBSS) by pooling DRG from L1–L6 or separately by segmental level where needed (L1, L5). Isolated DRG were transferred to FCS-coated tubes using siliconized transfer pipettes, prior to dissociation in 0.25% trypsin/0.1% Collagenase H solution for 10 minutes at 37°C. Excess ice cold HBSS was added before centrifugation (7 minutes, 800 rpm) and resuspension in 1 ml ice cold HBSS. Cells were dissociated by trituration using fire polished Pasteur pipettes to generate single cell suspension, and passed through 40 µm gauze filters to eliminate remaining cell aggregates. For Fluorescent Activated Cell Sorting (FACS), a MoFlo (DAKO) high-speed 4-way cell sorter with a 3-laser setup (two water-cooled Coherent Enterprise II lasers Model610 and Model653, and one air-cooled Spectra Physics Helium-Neon laser) was used to separate GFP^on^ from GFP^off^ cells. Suspended cells were first gated for fluorescence, using an excitation wavelength for green fluorescence of 488 nm, detected with a HQ515/30 bandpass filter. Using this approach, we gated for the GFP^on^ population, which was subsequently gated for size by measuring forward and side scatter. A 100 µm nozzle was used at 20 psi and sorted cells were collected in 1 ml Eppendorf tubes filled with 50 µl Trizol to allow for subsequent RNA isolation. Average cell number used per data point was 350 for all experiments, except for L1/L5 p0 data sets for which 100 cells were collected per sample.

### Affymetrix Gene Expression Profiling Experiments and Statistical Analysis

Affymetrix gene expression profiling experiments were performed by the FMI Genomics Facility essentially as described [26]. Briefly, RNA was extracted from isolated cells with the Pico Pure RNA Isolation kit (KIT0204, Life Technologies). Preparation of *in vitro* transcription (IVT) products was performed according to Affymetrix protocols (GeneChip Expression Analysis Technical manual, Rev. 5) with minor modifications. The fragmented cRNA (15 µg) was used in a hybridization cocktail containing spiked controls (Affymetrix). A total of 200 µl of this hybridization cocktail was hybridized at 45°C for 16 hours to GeneChip Mouse Genome 430 2.0 Arrays (Affymetrix). Following hybridization, the arrays were processed using a GeneChip Fluidics Station 400 according to recommended protocols (FS450_0001, Affymetrix). Fluorescent images of arrays were captured using the GeneChip Scanner 3000 7G (Affymetrix), and image data were acquired and analyzed using the Affymetrix GCC Scan Control v. 3.0.0.1214. For all experiments shown in this study, 2–3 replicates were carried out for each data point. Raw data from CEL files was loaded into R using the R (v2.9) / Bioconductor (v1.6) package affy. Data was normalized with the rma() function and present/absent calls were calculated using MAS5 normalization as implemented in the affy package. Normalized data was saved in .abs files for import into Genedata Analyst 2.2. Lists of differentially expressed genes were generated either using the t-test function (p ≤ 0.02) or the n-Way ANOVA function (p ≤ 0.02) as implemented in Genedata Analyst 2.2. In addition, we applied a cut-off of an at least 1.5 to 2 fold for changes of expression levels analyzed as indicated throughout the study. Heatmaps and accompanying z-scores were generated in R using heatmap function of the gplot package. Annotation files Mouse430_2.na24.annot.csv and Mouse430_2.na24.annot.csv for the GeneChip Mouse Genome 430 2.0 array were downloaded from www.affymetrix.com and used in Analyst 2.2. Significant difference between ≥4 groups was determined by 2-way ANOVA as implemented in Genedata Analyst 2.2, with a cut-off value of p≤0.02. Statistical significant difference between two groups was determined by t-test as implemented in Genedata Analyst 2.2 and defined as: *p≤0.05, **p≤0.01 and ***p≤0.001. The entire dataset was uploaded to the Gene Expression Omnibus Database (Accession Number GSE38074) at NCBI for public access (http://www.ncbi.nlm.nih.gov/geo/query/acc.cgi?acc=GSE38074).

### *In situ* Hybridization, qPCR and Immunohistochemistry

*In situ* hybridization experiments were performed as previously described [17]. *Gabrg1* and *Grm3* sequence for probes was amplified from adult brain cDNA using information from the Allen Brain webpage (*Gabrg1* riboprobe ID RP_080225_03_F04; forward: 5′-GCTACATCTGTGAGAGGAGGCT-3′; reverse 5′-ATAGTGAAGCATGTTACGCCCT-3′; *Grm3* riboprobe ID RP_081002_02_D05; forward: 5′-CCTCCTTTGCCATCTGTTATTC-3′; reverse 5′-GCCTAGAAAGTTGTAGCACATCA-3′). The following additional *in situ* hybridization probes were used in this study: *Etv1* [17], *Esrrg* [27], and *Pth1r* (Image clone accession number BC051981). Most probes used also consistently hybridized to bone tissue adjacent to DRG, which was not further analyzed here, and in most cases can be considered to reflect background signal. For qPCR experiments, adult DRG were collected and homogenized in Trizol using a glass homogenizer and a QIAshredder spin column (QIAGEN). Samples were then purified using the RNeasy mini kit (QIAGEN). Quantitative PCR was performed using Platinum Sybr Green Super mix UDG with ROX (Invitrogen no. 11790-01K) and *Gabrg1* signal was normalized to GAPDH signal. The following primers were used for qPCR amplification: *Gabrg1* (forward: 5′-CATAAACATGGAGTATACAATAG-3′; reverse: 5′-GAGTTCCTGAAGAAAGTGTC-3′) and GAPDH (forward: 5′-CATAAACATGGAGTATACAATAG-3′; reverse: 5′-GAGTTCCTGAAGAAAGTGTC-3′). Antibodies used in this study were: chicken anti-GFP (Invitrogen), guinea pig anti-Pvalb (Chemicon), guinea pig anti-vGlut1 (Chemicon), goat anti-ChAT (Chemicon), goat anti-LacZ (Biogenesis), goat anti-TrkC (R&D), rabbit anti-Cx36 (Invitrogen) [28], rabbit anti-GFP (Invitrogen), rabbit anti- Pvalb (Swant), and rabbit anti-Runx3 [29]. Spinal cords and DRG were sectioned and processed for immunohistochemistry as previously described [17] and images were acquired with an Olympus confocal (FV1000).

### Running Wheel Experiments with Adult Mice

Five weeks old male C57Bl6 mice were maintained in cages with running wheels for five weeks and running distances were measured with a counter attached to the running wheel. Littermate control mice were kept in identical cages without running wheels. Average distances run for the mice used for Affymetrix gene expression studies were approximately 5 km/24 hours. At the end of the experiment, L1 and L5 DRG were dissected and processed for RNA isolation and gene expression profiling.

## Results

### Selective Purification of Proprioceptors

**Figure 1:**
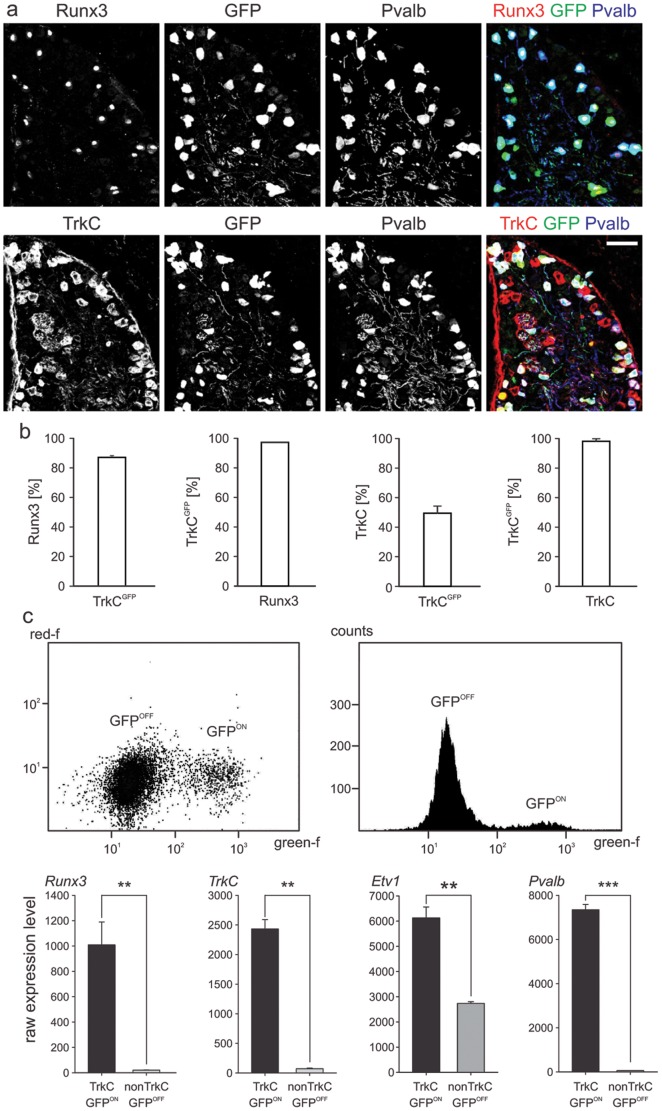
*TrkC^GFP^* BAC line shows enriched expression in proprioceptors. (a) Immunohistochemisty showing expression overlap between GFP and genes expressed by proprioceptors in p0 L5 DRG of *TrkC^GFP^* BAC transgenic mouse line (top row: Runx3 and Pvalb; bottom row: TrkC and Pvalb; overlay of all three channels shown to the right; scale bar = 60 µm). (b) From left to right: Quantification of percentage of Runx3^on^ neurons co-expressing the TrkC^GFP^ allele (86.4% ± SEM), percentage of TrkC^GFPon^ cells co-expressing Runx3 (97.6% ± SEM; of neurons with Isl1^on^ nucleus on section), percentage of TrkC^on^ cells co-expressing TrkC^GFP^ (49.6% ± SEM) and of percentage of TrkC^GFPon^ cells co-expressing TrkC (grey bar: 98.5% ± SEM). Data from n = 4 p0 *TrkC^GFP^* mice; total >20 L5 DRG sections. (c) Example of FACS scatterplot (left) and histogram (right) on dissociated lumbar DRG cells isolated from p0 *TrkC^GFP^* mice gated by fluorescence (GFP^off^ and GFP^on^ cells are indicated; red-f: red fluorescence; green-f: green fluorescence). Four examples of genes (*Runx3*, *TrkC*, *Etv1*, *Pvalb*) with known enriched expression in proprioceptors (TrkC^GFPon^) when compared to non-proprioceptors (TrkC^GFPoff^). Each panel shows Affymetrix expression values to the left (y-scale raw expression values; ± SEM).

To isolate genes with enriched expression in DRG proprioceptors, we made use of a GENSAT BAC transgenic mouse line in which the expression of enhanced green fluorescent protein (GFP) is controlled by genomic regulatory elements of the neurotrophin receptor TrkC (*TrkC^GFP^*) [23]. We first characterized transgene expression in relation to known markers of DRG neuron subpopulations with particular emphasis on proprioceptors. The expression of the Runt domain transcription factor Runx3 is highly restricted to proprioceptive DRG neurons [29], [30], prompting us to determine the overlap between Runx3 and GFP expression in *TrkC^GFP^* transgenic mice. We found that in p0 lumbar DRG, >95% of GFP^on^ DRG neurons co-express Runx3, and conversely, also most Runx3^on^ neurons are associated with GFP expression (Fig. 1a, b). A similar degree of overlap was also observed for the calcium binding protein Parvalbumin (*Pvalb*), a well-known marker of proprioceptors [17] (Fig. 1a). Quantitative analysis of overlap between TrkC and GFP expression in p0 lumbar DRG of *TrkC^GFP^* transgenic mice showed that >95% of GFP^on^ DRG neurons co-express TrkC (Fig. 1b). In summary, *TrkC^GFP^* mice show highly selective transgene expression in Runx3^on^/ Pvalb^on^/TrkC^on^ proprioceptors, making them the ideal tool for purification and transcriptional profiling of proprioceptors.

We next dissociated p0 lumbar DRG (segmental levels lumbar L1–L6) from *TrkC^GFP^* mice into single cell suspensions, and separated GFP^on^ proprioceptor from GFP^off^ non-proprioceptor populations by Fluorescent Activated Cell Sorting (FACS) (Fig. 1c). To determine genome-wide transcription differences between these two populations, we isolated and amplified RNA of these populations for subsequent Affymetrix microarray experiments. To get a first impression of the efficiency and reliability of this approach in detecting genes with proprioceptor-enriched gene expression, we analyzed expression profiles of four genes with previously known association to proprioceptors. Confirming selective GFP expression in proprioceptors, the expression of *Runx3* and *TrkC* was highly enriched in the GFP^on^ population when compared to the non-proprioceptor GFP^off^ population (Fig. 1c), and *Pvalb* as well as the ETS transcription factor *Etv1* also scored as highly enriched (Fig. 1c).

**Figure 2:**
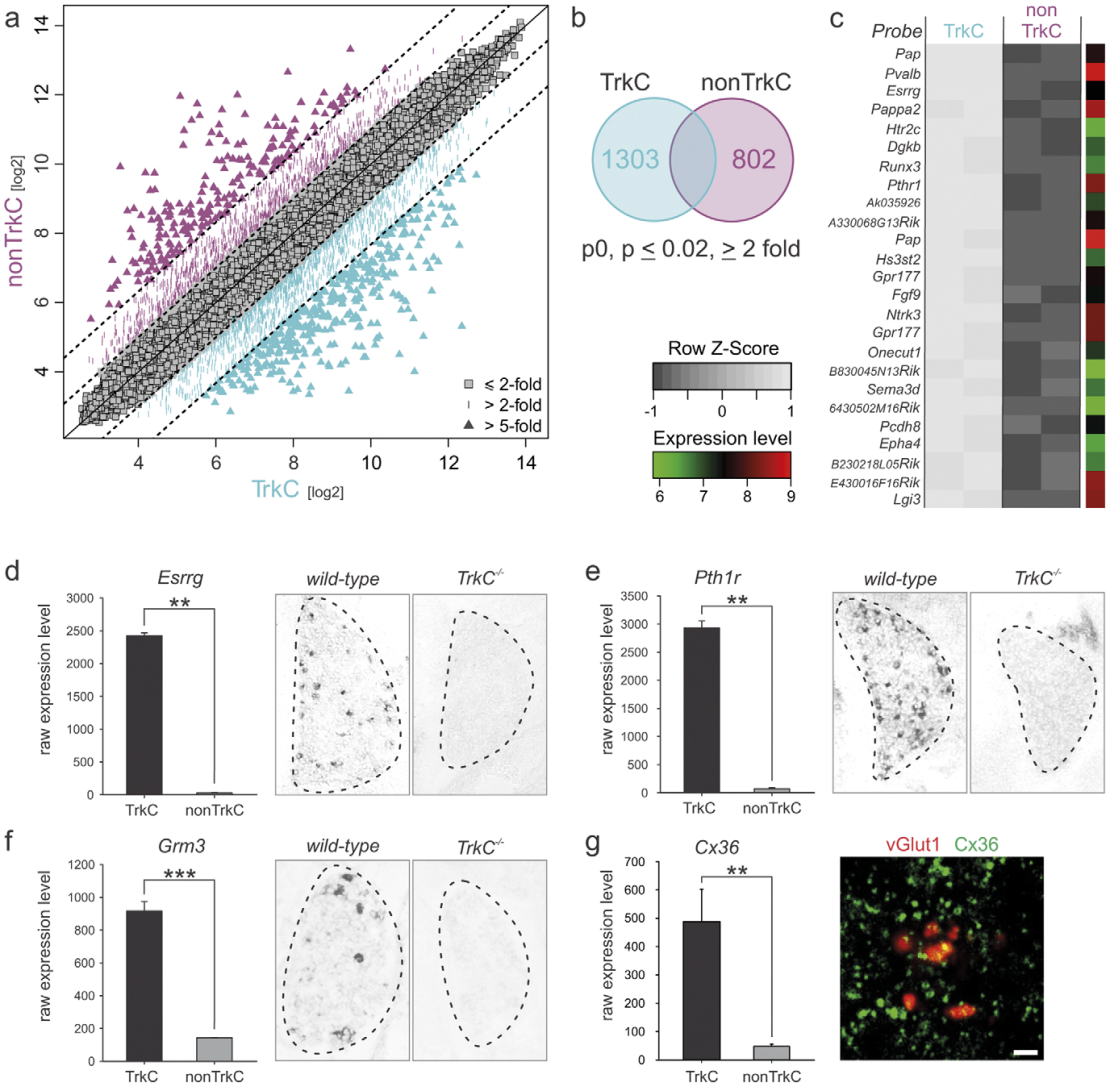
Isolation of genes with enriched expression in proprioceptors. (a) Affymetrix gene expression profiling data showing probes enriched in TrkC^GFPon^ proprioceptors and TrkC^GFPoff^ non-proprioceptors isolated from p0 mice. Diagonal lines indicate cut-off for probes with expression values >5 fold change (outermost dotted lines), >2 fold change (middle dotted lines) and ≤2 fold change (grey squares around central diagonal line). TrkC^GFPon^ proprioceptor data points with enrichment ≥2 fold are displayed in turquoise and TrkC^GFPoff^ non-proprioceptors with the same criteria in purple. (b) Venn diagram illustrating the number of probes enriched ≥2 fold in proprioceptors and non-proprioceptors isolated from p0 mice respectively. (c) Analysis of the 25 probes with highest fold changes displayed in detail. Values of two samples of each p0 TrkC^GFPon^ proprioceptors (left) and TrkC^GFPoff^ non-proprioceptors (right) are shown. Grey scale values represent row z-score values and log unit average expression values are shown to the right of each probe (scales plotted bottom left). Probe names are displayed to the left of each row. (d–g) Four examples of individual genes with highly enriched expression in proprioceptors (TrkC) when compared to non-proprioceptors (non-TrkC) are displayed. Each panel shows raw Affymetrix expression values to the left (y-scale expression values; ±SEM) and verification by either *in situ* hybridization on wild-type and *TrkC* mutant lumbar DRG sections (d–f; scale bar = 50 µm) or immunohistochemistry in ventral spinal cord lamina IX (g; green: Cx36; red: vGlut1; scale bar = 2 µm) to the right.

To perform a quantitative genome-wide analysis of gene expression differences, we used a significance threshold of p≤0.02 and an enrichment factor of ≥2 fold between TrkC^GFPon^ and TrkC^GFPoff^ populations. Using these criteria, we found that 1303 of ∼45 K expressed probes on the Affymetrix chip set used were enriched in TrkC^GFPon^ proprioceptors, and conversely, 802 probes exhibited clear enrichment in the TrkC^GFPoff^ non-proprioceptor population (Fig. 2a, b). We next analyzed in more detail the 25 probes with the highest observed expression differences based on fold changes between TrkC^GFPon^ and TrkC^GFPoff^ populations, and found that these hits encompassed genes of various expression levels, indicating no particular bias towards a specific expression level as a contributing factor to enrichment (Fig. 2c). In addition, and in agreement with the high fold changes detected, z-score analysis reveals a strong deviation of the two populations from the distribution mean (Fig. 2c). Together, these findings demonstrate that many genes show enriched expression in proprioceptors.

To further probe the reliability of our data at the single gene level, we picked four genes not previously known to exhibit proprioceptor-enriched gene expression profiles. We verified the selective expression pattern of these genes either by immunohistochemical analysis or by *in situ* hybridization experiments on tissue from wild-type and *TrkC* mutant mice in which proprioceptors are eliminated at early developmental stages due to the absence of neurotrophin signaling essential for proprioceptor survival [25]. The orphan transcription factor estrogen related receptor *Esrrg*, with previously shown expression in gamma motor neurons in the ventral spinal cord [27], exhibited highly selective enrichment in proprioceptors by the Affymetrix gene expression profiles and *in situ* hybridization verified the complete absence of expression in *TrkC* mutant mice (Fig. 2d). Parathyroid hormone 1 receptor (*Pth1r*), a receptor with prominent role in bone formation [31] and currently unknown function in the nervous system, also exhibited highly enriched expression in proprioceptors within the DRG and displayed complete absence of expression in *TrkC* mutant mice (Fig. 2e). Metabotropic glutamate receptor 3 (*Grm3*), a gene with significant gene variant associations linked to memory performance in humans [32], revealed scattered cells within the DRG by *in situ* hybridization, a pattern absent in *TrkC* mutant mice (Fig. 2f). In contrast to *Esrrg* and *Pth1r* however, *Grm3* exhibited a much sparser labeling density within the DRG, indicating that its expression is confined to only a subset of proprioceptors. These findings demonstrate that our approach not only picks up genes expressed by all TrkC^on^ DRG neurons, but is sensitive enough to isolate genes with expression in proprioceptor subsets, a feature further exploited later in this study. Lastly, we also determined whether genes expressed by proprioceptors produce proteins transported to central synapses, exploiting the example of connexin 36 (*Cx36*), a gap junction protein with known neuronal expression and required for gap junction function [33]. Using an antibody to Cx36 [28], we determined whether proprioceptor terminals in the ventral spinal cord marked by the selective accumulation of vesicular glutamate transporter 1 (vGlut1) [34] exhibit colocalization with the gap junction protein Cx36. We found association of vGlut1^on^ proprioceptive terminals in spinal lamina IX with Cx36^on^ signal (Fig. 2g). These findings suggest that gap junction proteins are present and might play a role at proprioceptive central synapses, a view supported by recent functional evidence demonstrating that *Cx36* mutant mice exhibit electrophysiologically detectable defects in presynaptic inhibition [28].

Together, these findings demonstrate the reliability of our Affymetrix gene expression experiments in isolating genes with highly enriched expression in proprioceptive afferents when compared to non-proprioceptor populations, and allow us to exploit this method further to study the regulation of these genes by perturbation of peripheral neurotrophic factor signaling.

### Elimination of NT3 Profoundly Alters Proprioceptor Gene Expression

We next determined the effect of NT3 elimination on gene expression in DRG neurons. Since *NT3* mutant mice exhibit pronounced DRG neuron cell death at early developmental stages due to an essential role of NT3 in promoting neuronal survival [13], [20], we made use of the observation that concurrent elimination of the proaptototic gene *Bax* in mice circumvents DRG neuron cell death and allows studying a role of NT3 other than for the regulation of neuronal survival [16]. Using this insight, we compared genome-wide expression profiles between proprioceptors and non-proprioceptors isolated by virtue of the *TrkC^GFP^* BAC allele, in each of the three genetic backgrounds of wild-type, *NT3^−/−^Bax^−/−^*, and *Bax^−/−^* DRG. This three-way comparison would allow us to avoid focusing on genes affected in expression solely due to *Bax* mutation.

**Figure 3:**
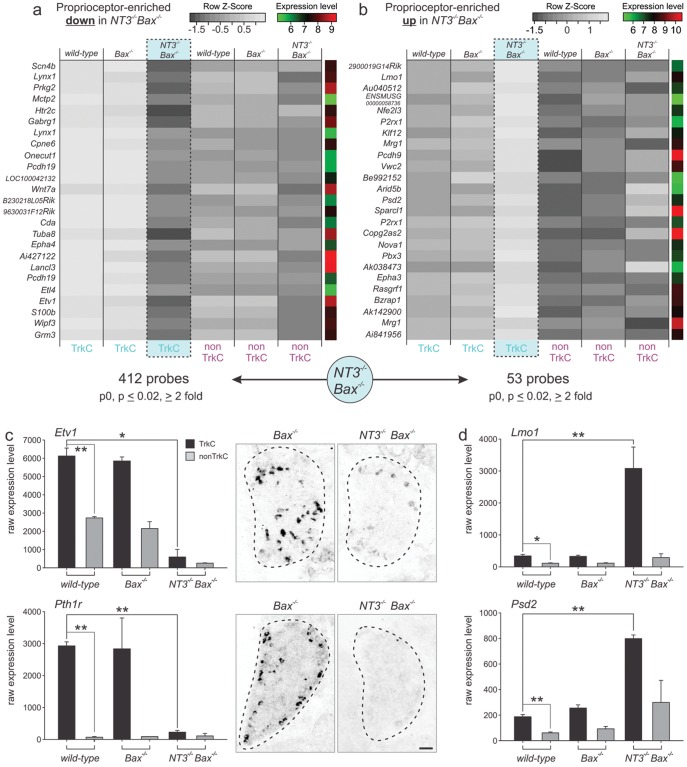
NT3 deletion alters proprioceptor gene expression. (a, b) Analysis of 25 downregulated (a) or upregulated (b) probes with highest fold changes in *NT3^−/−^Bax^−/−^* mice relative to wild-type is displayed. Average values of p0 TrkC^on^ proprioceptors (left; TrkC) and TrkC^off^ non-proprioceptors (right; non-TrkC) isolated from wild-type, *Bax^−/−^* and *NT3^−/−^Bax^−/−^* mice are shown. Grey scale values represent row z-score values and log unit average expression values are shown to the right of each probe (scales plotted top right of each panel). Probe names are displayed to the left of each row. The number of probes regulated in proprioceptors (p≤0.02; regulation ≥2 fold) is shown below the plots. (c, d) Detailed expression analysis of two individual genes downregulated (*Etv1* and *Pth1r*) and two genes upregulated (*Lmo1* and *Psd2*) in proprioceptors of *NT3^−/−^Bax^−/−^* but not in *Bax^−/−^* mice is shown (Affymetrix analysis: y-scale displays raw expression values; ± SEM). For *Etv1* and *Pthr1*, also *in situ* hybridization results on p0 lumbar DRG of in *Bax^−/−^* and *NT3^−/−^Bax^−/−^* mice are displayed to the right (Scale bar = 50 µm).

Comparison of gene expression data revealed that 412 of the wild-type proprioceptor enriched probe sets were also significantly downregulated in proprioceptors of *NT3^−/−^Bax^−/−^* but not affected in *Bax^−/−^* mice (Fig. 3a; ANOVA p≤0.02; regulation ≥2 fold). In contrast, a comparatively small fraction of genes (53 probes) with proprioceptor-enriched expression profile were upregulated in *NT3^−/−^Bax^−/−^* proprioceptors (Fig. 3b; ANOVA p≤0.02; regulation ≥2 fold). To probe the reliability of these results, we determined the expression profiles of several individual genes in more detail. We first analyzed the expression profiles of *Etv1*, with previously described regulation by peripheral NT3 [16]. We found that *Etv1* expression was enriched in proprioceptors of both wild-type and *Bax^−/−^* mice, much in contrast to the observed expression in *NT3^−/−^Bax^−/−^* mice, where *Etv1* expression was very low, a pattern which was also confirmed by *in situ* hybridization on DRG sections (Fig. 3c). Moreover, *Pth1r*, which scored amongst the genes with highest proprioceptor enrichment (Fig. 2c, e), also showed dramatic downregulation in *NT3^−/−^Bax^−/−^* mice by *in situ* hybridization (Fig. 3c). Conversely, the genes encoding the Lim-domain containing protein Lmo1 and Pleckstrin/Sec7 domain containing protein 2 (Psd2) exhibited enrichment in proprioceptors in wild-type and *Bax^−/−^* mice, but striking upregulation in *NT3^−/−^Bax^−/−^* proprioceptors (Fig. 3d). We also mined gene expression profiles unchanged in proprioceptors of *NT3^−/−^Bax^−/−^* mice and found genes with previously studied function in proprioceptor differentiation such as the receptor for NT3 itself (*TrkC*) (data not shown). Together, these findings demonstrate that genetic elimination of *NT3* affects gene expression of a selective subset of genes with enriched expression in proprioceptors, and of the genes affected, most genes with significant expression changes are downregulated by developmental genetic deprivation of NT3.

### Surplus Skeletal Muscle NT3 Expression Alters Proprioceptor Gene Expression

Since complete genetic elimination of NT3 by virtue of studying *NT3^−/−^Bax^−/−^* mice revealed pronounced effects on gene expression in proprioceptors, we next sought to determine whether raising NT3 levels in skeletal muscles would also affect proprioceptor gene expression. Previous work demonstrated that altering NT3 levels genetically to abnormally high values by transgenic expression of NT3 using the skeletal muscle promoter myosin light chain (*mlc*) leads to a dramatic breakdown of the specificity in central connectivity between group Ia proprioceptors and motor neurons [21], [22], suggesting that accurate muscular NT3 levels influence central connectivity by retrograde signaling.

**Figure 4:**
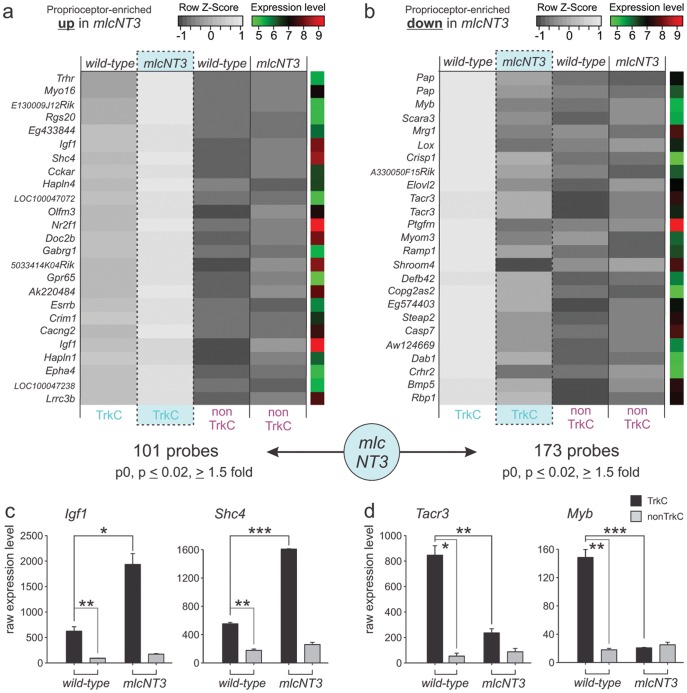
Surplus skeletal muscle NT3 alters proprioceptor gene expression. (a, b) Analysis of 25 upregulated (a) or downregulated (b) probes in *mlc^NT3^* mice is displayed. Average values of p0 TrkC^on^ proprioceptors (left; TrkC) and TrkC^off^ non-proprioceptors (right; non-TrkC) isolated from wild-type and *mlc^NT3^* mice are shown. Grey scale values represent row z-score values and log unit average expression values are shown to the right of each probe (scales plotted top right of each panel). Probe names are displayed to the left of each row. The number of probes regulated in proprioceptors (p≤0.02; regulation ≥1.5 fold) is shown below the plots. (c, d) Detailed expression analysis of two individual genes upregulated (*Igf1* and *Shc4*) and two genes downregulated (*Tacr3* and *Myb*) in proprioceptors of *mlc^NT3^* mice is shown (Affymetrix analysis: y-scale displays raw expression values; ±SEM).

A genome-wide analysis of gene expression differences showed 101 probe sets with significant upregulation in proprioceptors of *mlc^NT3^* mice compared to wild-type (Fig. 4a; ANOVA p≤0.02; regulation ≥1.5 fold). Conversely, 173 probe sets scored as significantly downregulated in proprioceptors of *mlc^NT3^* mice (Fig. 4b; ANOVA p≤0.02; regulation ≥1.5 fold). Again, these gene expression changes could be analyzed at the level of individual genes (Fig. 4c, d, data not shown), where for example the genes encoding for insulin-growth factor 1 (*Igf1*) and Src homology 2 domain containing family member 4 (*Shc4*) were upregulated in *mlc^NT3^* mice (Fig. 4c), whereas Tachykinin receptor 3 (*Tacr3*) and Myoblastosis oncogene (*Myb*) were downregulated (Fig. 4d). Interestingly, Igf1 had previously been identified as upregulated by the application of NGF and BDNF to distal axons of cultured DRG neurons [35]. In *mlc^NT3^* mice, as was the case for *NT3^−/−^Bax^−/−^* mice, we observed many genes with enriched expression in wild-type proprioceptors, but without perturbation in expression by raising peripheral NT3 levels (data not shown). Together, these findings demonstrate that not only complete elimination of *NT3* affects gene expression in proprioceptors, but also raising NT3 levels in skeletal muscles leads to profound and significant gene expression changes in proprioceptors, pointing to possible molecular entry points to understand the observed central connectivity defects in *mlc^NT3^* mice [22]. Not unexpectedly though, expression changes detected in *mlc^NT3^* mice were less dramatic and numerous than in *NT3^−/−^Bax^−/−^* mice.

### Anti-correlative Proprioceptor Gene Expression Profiles by Genetic *NT3* Manipulation

**Figure 5:**
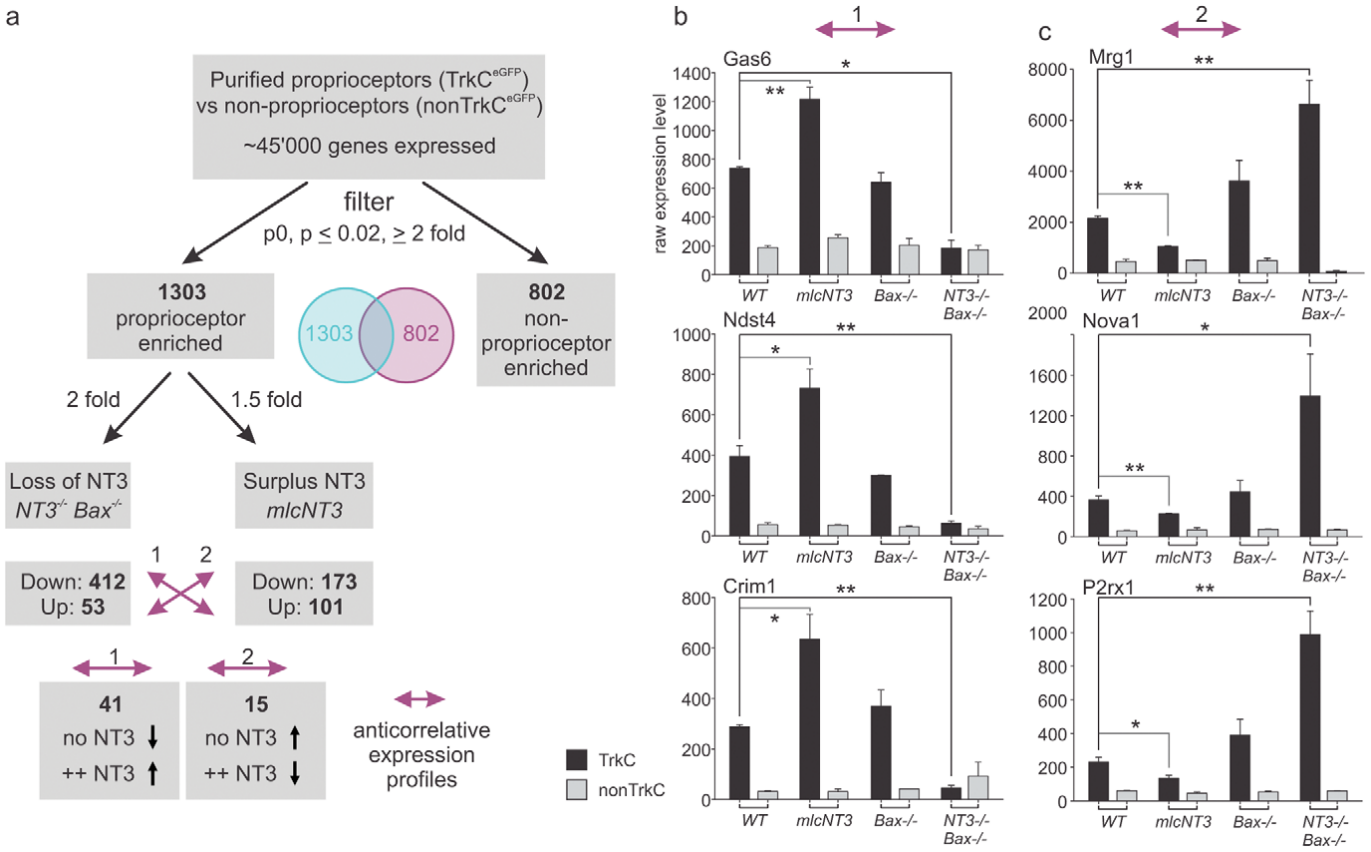
Genes with anticorrelation in NT3 regulation profiles. (a) Flow diagram to illustrate analysis of gene expression data extracted from proprioceptors and non-proprioceptors of various mouse strains. To isolate genes with anticorrelative expression profile, genes with expression changes in opposing directions in proprioceptors of *NT3^−/−^Bax^−/−^* mice and *mlc^NT3^* mice were isolated (purple arrows 1 and 2). (b, c) Detailed expression analysis of three individual genes following anticorrelation scheme 1 (*Gas6*, *Ndst4*, and *Crim1*) and three genes following scheme 2 (*Mrg1*, *Nova1*, and *P2rx1*). Affymetrix analysis: y-scale displays raw expression values; ± SEM.

We next performed an analysis of genes with proprioceptor-enriched expression and regulated by NT3 signaling using more stringent criteria, by combining the two strategies of genetic manipulations, elimination of NT3 in *NT3^−/−^Bax^−/−^* mice and rise of NT3 in *mlc^NT3^* mice. We reasoned that genes with anticorrelative expression profiles in proprioceptors isolated from these mice would likely be those most perceptive in sensing NT3 level changes, and therefore react in opposite directions by adjusting expression in response to altered peripheral signaling. Since most proprioceptor-genes with significant expression changes in *NT3^−/−^Bax^−/−^* mice were downregulated (probes: 412 down vs 53 up; Fig. 5a), we were most interested in which ones of these genes were upregulated in *mlc^NT3^* mice. In this anti-correlative analysis, 41 probes matched these criteria (Fig. 5a, b; 41/101 probes; Table S1). These findings indicate that ∼40% of all genes upregulated upon rising peripheral NT3 were regulated in the opposite direction upon complete genetic elimination of NT3, whereas ∼90% of genes downregulated in *NT3^−/−^Bax^−/−^* mice were not altered in *mlc^NT3^* mice. The opposite anticorrelative analysis identified 15 probes with increased expression in *NT3^−/−^Bax^−/−^* mice and decrease in *mlc^NT3^* mice (Fig. 5a, c; Table S1).

### *Gabrg1* Exhibits Anticorrelative NT3 Regulated Expression Profile in Proprioceptors

**Figure 6:**
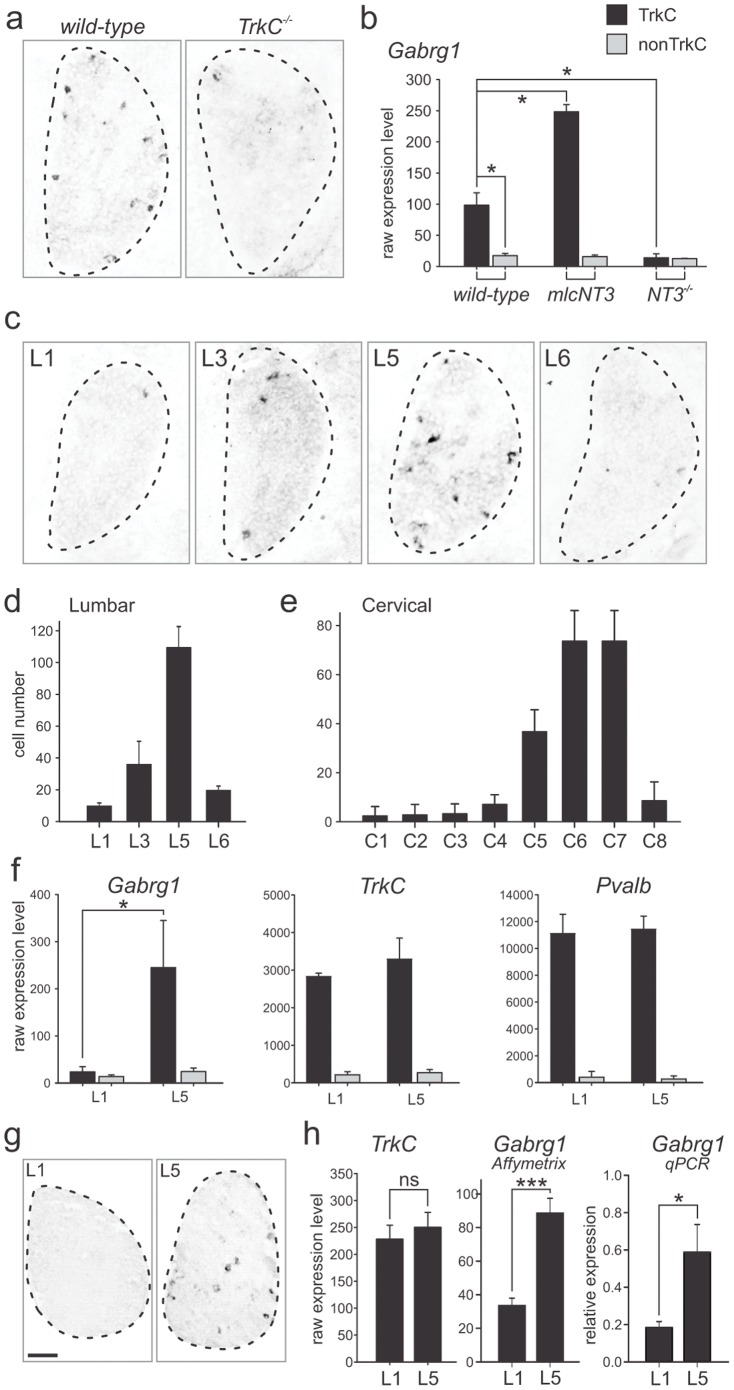
*Gabrg1* expression in proprioceptors in rostro-caudal gradient and regulated by NT3. (a) *In situ* hybridization experiments demonstrating downregulation of *Gabrg1* expression in p0 *TrkC* mutant L5 DRG when compared to wild-type. (b) Affymetrix expression value analysis of *Gabrg1* in proprioceptors (TrkC) and non-proprioceptors (nonTrkC) of wild-type (left), *mlc^NT3^* (middle) and *NT3^−/−^Bax^−/−^* (right) mice (y-scale displays raw expression values; ±SEM). Note selective upregulation of *Gabrg1* in proprioceptors of *mlc^NT3^* and downregulation in *NT3^−/−^Bax^−/−^* mice compared to wild-type values, in absence of gene expression changes in non-proprioceptors. (c–e) *In situ* hybridization analysis of *Gabrg1* expression on p0 wild-type DRG, displaying representative images from lumbar levels L1, L3, L5 and L6 (c) and quantification of cell numbers at lumbar (d) and cervical (e) rostro-caudal levels (n = 3 animals; ±SEM). (f) Affymetrix expression value analysis of *Gabrg1*, *TrkC*, and *Pvalb* at L1 and L5 (y-scale displays raw expression values; ±SEM). (g, h) Analysis of *Gabrg1* expression in adult DRG by *in situ* hybridization (g: L1 and L5; scale bar = 80 µm), Affymetrix gene expression values (h; n = 3; ±SEM), and quantitative PCR (h; n = 3; ±SEM). In comparison, Affymetrix expression values of *TrkC* are not significantly different between L1 and L5 adult DRG (h; n = 3; ±SEM).

Of the list of genes regulated in an anticorrelative manner in proprioceptors of *NT3* signaling mutants, *Gabrg1*, a gene encoding a subunit of the GABAA receptor family [36], [37], [38] showed a very striking expression profile across all mutants. *In situ* hybridization on p0 DRG showed the almost complete absence of *Gabrg1* expression in *TrkC* mutant DRG when compared to wild-type (Fig. 6a), as well as an increase in cells expressing *Gabrg1* in *mlc^NT3^* mice (Fig. S1). Assessing individual Affymetrix microarray profiles of *Gabrg1*, we found that its expression was nearly completely downregulated in *NT3^−/−^Bax^−/−^* mice while increased significantly and selectively in proprioceptors of *mlc^NT3^* mice, whereas expression in non-proprioceptor populations was not altered in any of the mutants and was at low levels throughout (Fig. 6b).

Performing *in situ* hybridization experiments, we noticed an unequal density of *Gabrg1^on^* cells along the rostro-caudal axis. Since lumbar DRG were pooled for Affymetrix microarray analysis, this observation prompted us to carefully quantify the number of cells detected at different segmental levels by *in situ* hybridization. We found a progressive increase in the number of *Gabrg1^on^* cells from L1 toL5, and a drop at the L6 level (Fig. 6c, d). In addition, quantitative assessment of *Gabrg1^on^* cell number across cervical level DRG C1-C8 revealed a similar gradient in expression as at lumbar levels, with low numbers of *Gabrg1^on^* cells at C1–C4, and gradually increasing numbers at C5–C7 (Fig. 6e). To assess whether this rostro-caudal number increase is independent of the higher number of TrkC^on^ neurons at L5 than L1 lumbar DRG, we isolated p0 *TrkC^GFPon^* proprioceptors separately from L1 and L5 DRG by FACS and acquired genome-wide Affymetrix expression profiles. We found that while *TrkC* and *Pvalb* expression levels in *TrkC^GFPon^* proprioceptors are constant across these segmental levels and consistently highly enriched compared to their expression in non-proprioceptors, *Gabrg1* expression levels increase between L1 and L5 selectively in proprioceptors (Fig. 6f). These findings demonstrate that the observed increase in *Gabrg1^on^* cell number across rostro-caudal lumbar levels is independent of *TrkC^GFPon^* proprioceptor numbers.

To assess whether the subset restricted expression profile of *Gabrg1* is maintained in adult DRG, when GABA receptors are predicted to have a functional role, we first carried out *in situ* hybridization experiments at L1 and L5 DRG of adult mice (p35). We found that also in the adult, *Gabrg1* expression in lumbar DRG was confined to sparsely scattered cells and exhibited rostro-caudal density differences between L1 and L5 (Fig. 6g). These L1–L5 segmental differences were also observed at the level of entire DRG by using Affymetrix gene transcription profiling or quantitative PCR, comparing expression values between L1 and L5, whereas *TrkC* expression values in the same samples did not differ between these segmental levels (Fig. 6h). Together, these findings provide evidence that *Gabrg1* expression in lumbar DRG exhibits pronounced rostro-caudal differences also at adult stages, a pattern likely maintained from early postnatal stages.

### Regulation of Gene Expression in Sensory Neurons by Voluntary Running

**Figure 7:**
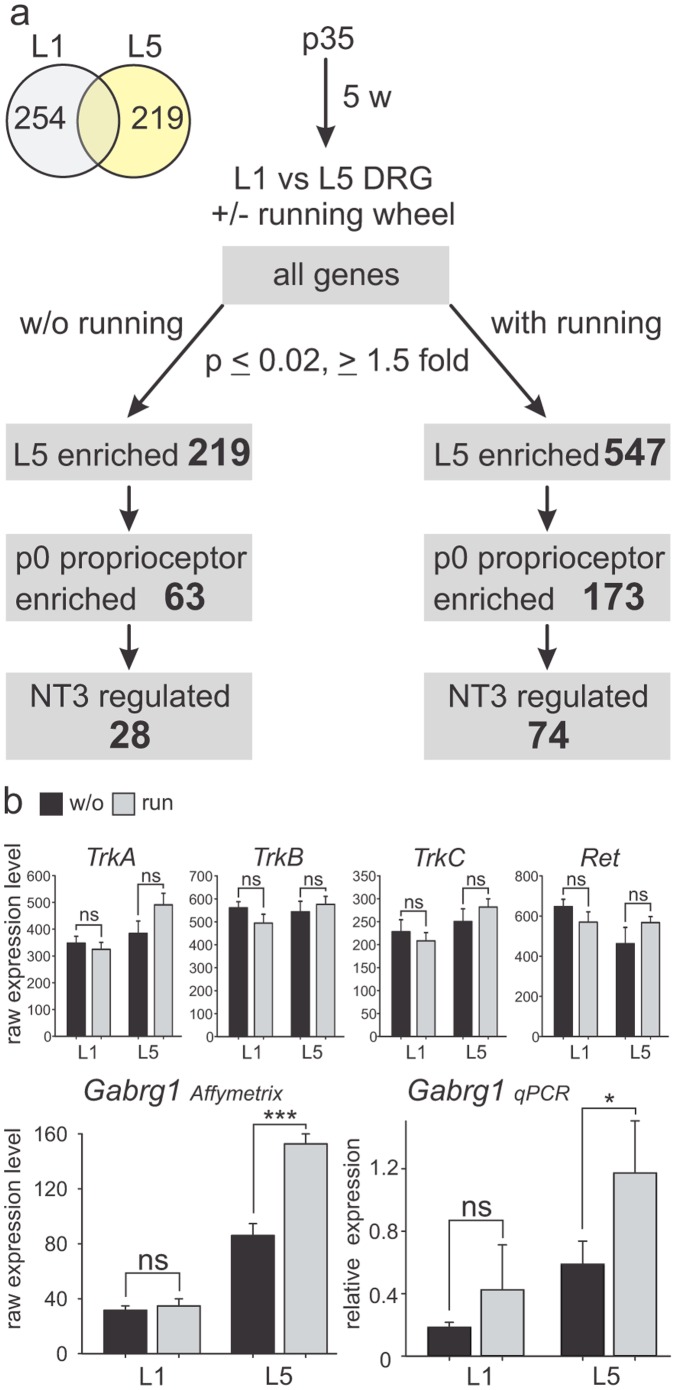
Voluntary running influences gene expression and *Gabrg1*. (a) Analysis of the influence of voluntary running wheel usage on gene expression in L1 and L5 adult DRG (p≤0.02; regulation ≥1.5 fold regulated). L5 enriched genes in each of the two conditions were compared to p0 proprioceptor enriched gene list and NT3-regulated genes in proprioceptors. Number of probes identified is indicated in respective grey boxes. (b) Analysis of *TrkA*, *TrkB*, *TrkC*, *Ret*, and *Gabrg1* expression in adult L1 and L5 DRG of mice with (grey bars) and without (w/o: black bars) running wheel experience, using Affymetrix gene expression analysis (left; n = 3; ±SEM) or quantitative PCR for *Gabrg1* (right; n = 3; ±SEM).

*Gabrg1* is regulated by NT3 signaling and pronounced expression differences were detected at birth in mutants genetically eliminating NT3 or raising NT3 levels. These findings prompted us to probe whether there may also be conditions inducing transcriptional changes in adult DRG neurons, in particular upon implementation of behavioral protocols such as increased physical exercise. For this purpose, p35 mice were kept in cages with running wheels for 5 weeks before L1 and L5 DRG were isolated and compared by Affymetrix gene expression analysis (Fig. 7a). We found no significant changes in expression levels for *TrkA*, *TrkB*, *TrkC* and *Ret* analyzed at L1 and L5 in the two experimental groups (Fig. 7b). Since we were particularly interested in genes with L5-enriched expression patterns, we first assessed the number of probes with this profile in mice with and without running wheel experience (ANOVA p≤0.02; regulation ≥1.5 fold). We found L5-enrichment for 219 probes in control mice, 547 probes in running wheel mice, and an overlap of 161 probes between the two samples (Fig. 7a). Since expression profiles were derived from whole DRG and purification of adult proprioceptors is technically not feasible, we next asked how many probes with higher expression values at L5 also scored as proprioceptor-enriched using the data from the p0 analysis. For mice without running wheel experience, 63/219 probes scored as p0 proprioceptor enriched (p≤0.02; regulation ≥1.5 fold), and 173/547 probes expressed with L5 enrichment in DRG isolated from mice with running wheel experience were also proprioceptor-enriched in p0 DRG (Fig. 7a). Lastly, we analyzed how many of these probes were also amongst the cohort of genes regulated by NT3 signaling in p0 proprioceptors. We found that about half the probes with p0 proprioceptor enriched expression profiles also scored as p0 NT3 signaling-regulated (Fig. 7a; without running: 28/63 probes; with running wheel: 74/173 probes). Amongst the 74 NT3 regulated genes, *Gabrg1* exhibited a very distinct Affymetrix gene expression profile. Running wheel experience at the L1 level did not significantly alter *Gabrg1* expression, but at the L5 segmental level, showed a significant increase in expression detected at the whole DRG level, a feature that was also confirmed using quantitative PCR (Fig. 7b). Together, these findings support the notion that *Gabrg1* is not only regulated by peripheral retrograde signaling in subpopulations of DRG neurons at embryonic and neonatal stages, but its expression can still be modulated by behavioral interventions in the adult. In these behavioral experiments however, we have no evidence that upregulation is directly related to changes of NT3 levels in skeletal muscles, although it is interesting to note that a recent study demonstrated increased NT3 protein levels in hindlimb muscles upon physical exercise [39].

## Discussion

We used genome-wide gene expression profiling to determine the transcriptional consequences of NT3 signaling in proprioceptors. We found that the expression of many proprioceptor-enriched genes is dramatically regulated by genetic elimination of NT3. Combined with analysis of mice with surplus NT3 expression in skeletal muscles, we identify a specifically anticorrelated gene subset reacting in opposite directions to lower or higher NT3 levels in proprioceptors. We discuss our findings in the context of gene expression profiling experiments on neuronal subpopulations, work on retrograde signaling mechanisms involved in neuronal subpopulation specification, and possible roles for the regulation of synaptic signaling components in sensory circuit function.

### Identification of Subpopulation Specific Neuronal Gene Expression

Transgenic marking of proprioceptors in mice led to the isolation of a large number of genes with enriched expression in proprioceptors. The BAC transgenic line used in this study allowed us to separate TrkC^on^/Pvalb^on^/Runx3^on^ sensory neurons from TrkC^on^/Pvalb^off^/Runx3^off^ populations, which most likely represent mechanoreceptor subtypes innervating the skin [40], [41]. Neuronal purification was a prerequisite to success since proprioceptors only make up a minority of DRG neurons. Our observations add to a number of studies on gene expression profiling in the nervous system demonstrating that genes with highly enriched expression in defined neuronal subtypes making up small fractions of a sampled structure can be identified with much higher success rates upon neuronal purification [42]. Nevertheless, the proprioceptor population studied here does not represent a functionally unique population. It can be subdivided into group Ia/II afferents innervating muscle spindles and group Ib afferents targeting Golgi tendon organs in the periphery [8], [43], and both types of sensory organs in the muscle are innervated by *Pvalb* expressing sensory neurons [17]. In addition, individual proprioceptors only target one peripheral muscle and mechanisms controlling the establishment of axonal trajectories peripherally as well as the selection of specific central target neurons are currently unknown. The identification of genes expressed by proprioceptor subpopulations may provide an important entry point to study further diversification amongst proprioceptors, both with respect to functional subtypes as well as projection targets. Work on motor neurons provides evidence for dedicated molecular programs acting at the level of individual motor neuron pools projecting to distinct muscles as well as at the level of alpha- and gamma motor neuron subtypes innervating extra- and intrafusal muscle fibers [4], [27], [44]. Similar mechanistic insight is currently lacking for DRG subtypes, most likely due to the scattered neuronal cell body distribution, in contrast to the clustered organization of motor neurons. Our study demonstrates that this approach can successfully define genes with non-panproprioceptor expression profiles and provides a database to identify genes expressed in proprioceptors or subpopulations thereof for future studies.

### Cellular and Transcriptional Effects of Retrograde Signaling in Neuronal Subpopulations

The approach of isolating specific neuronal populations from mouse mutants allowed us to study the consequences of NT3 signaling on transcription in proprioceptors. NT3 represents an important signaling system not only for the control of proprioceptor cell survival [20], [25], [45], [46], [47], but from loss-of-function experiments preventing cell death phenotypes, it is also known to play a role in the establishment of the central trajectory of proprioceptors towards motor neurons [16]. In addition, overexpressing NT3 in skeletal muscles interferes with the establishment of specific synaptic connections between proprioceptors and motor neurons [22]. Our study provides insight into the transcriptional consequences of these NT3 level manipulations at the level of proprioceptors. We found that NT3 elimination in *NT3^−/−^Bax^−/−^* mice elicits a much more prominent downregulatory effect on gene expression than further raising expression in proprioceptors, suggesting that the normal role of NT3 is to mainly promote and/or enhance proprioceptor-specific gene expression. Changes in gene expression upon raising muscular NT3 levels were more modest, most likely reflecting the fact that not all genes have the ability to scale expression gradually in response to differing NT3 levels. It is the anticorrelative combinatorial analysis, which may point out specifically genes sensitive to gradual changes in NT3 levels. Our study is based on cell population analysis, thus not measuring gene expression values at the single cell level. For genes with expression patterns restricted to proprioceptor subsets, it is therefore difficult to predict whether gene expression changes observed in mutant backgrounds reflect expression changes at the level of individual cells or alterations in cell number expressing a certain gene. This question will have to be addressed using more refined methods such as single cell qPCR for individual genes in the future.

Our analysis focuses on the regulation of genes with enriched expression in proprioceptors. Nevertheless, retrograde NT3 signaling is not restricted to proprioceptors, nor is it limited to genes with proprioceptor-enriched expression patterns. This is not surprising since NT3 is known to have promiscuous signaling properties by binding to Trks other than TrkC as well as to p75 [10], [11], [13]. The same dataset can hence be analyzed to extract genes regulated by NT3 in non-proprioceptors using different filters and criteria for analysis. Moreover, genes with expression in proprioceptors and subsets of non-proprioceptors can also be identified. For example, the gene encoding the ETS transcription factor Etv1 is expressed in several DRG neuron subpopulations and regulated by NT3 in both proprioceptors and subsets of non-proprioceptors.

An emerging feature in studies on the influence of target-derived retrograde neuronal responses is the observation that postmitotic neurons are capable of adjusting their transcriptional programs in order to react to signals by changing their cellular phenotypes. These changes can become apparent by a variety of readouts. Studies on retrograde signaling pathways in NGF-responsive TrkA expressing DRG sensory neurons have provided evidence that NGF signaling promotes a switch to the emergence of non-peptidergic Ret-expressing sensory neurons [48], by virtue of inducing expression of Ret and GFRalpha coreceptors as well as other characteristic receptor genes in these neurons [49]. In the trigeminal somatosensory system, retrograde signaling by TGFbeta family members also acts to change transcriptional profiles in these whisker innervating sensory neurons, inducing the transcription factor Onecut2, which in turn is involved in the regulation of central projections [50]. The analysis performed on proprioceptors here lends support to the notion that retrograde signaling from the target area intersects in a very profound way with the transcriptional programs set up at stages before axons invade their targets.

### Synaptic Gene Expression Regulated by Neurotrophic Factors

A striking feature revealed by our analysis is the observation that some genes encoding synaptic components are regulated in proprioceptors by altering NT3 signaling. One of the most dramatic examples identified is the gene encoding the GABAA receptor subunit Gabrg1, a previously very poorly studied receptor subunit of currently unknown function [36], [37], [38]. What could be possible roles for postsynaptic receptor components in neurons with purely axonal extensions? It is well-known that central branches of DRG sensory neurons not only establish synaptic contacts with a variety of central spinal neurons themselves, but that their synaptic terminals also provide postsynaptic substrates for inhibitory axo-axonal synapses [51], [52], [53]. It is thought that through these axo-axonal synapses, presynaptic inhibition can selectively shunt incoming sensory information at the level of individual sensory synapses, thus preventing or significantly reducing a postsynaptic effect of sensory signals on spinal neurons [51], [52], [53]. Electrophysiological studies using pharmacological interventions show that GABAA receptors are involved in presynaptic inhibition [53], but which receptor components contribute and whether perhaps different sensory afferents assemble receptors of different composition is currently unknown. Moreover, receptors for neurotransmitters other than GABA are being considered for their involvement in this phenomenon [53], [54], suggesting that also other proprioceptor-enriched and in part NT3 regulated postsynaptic receptor components identified here (e.g. *Htr2c*) may be relevant for this process.

Our findings show that *Gabrg1* is expressed preferentially by proprioceptors of more caudal level DRG both at lumbar and cervical spinal levels. These spinal segments are known to innervate distal limb muscles, in contrast to more rostral segments innervating proximal limb muscles. It is currently unknown how such transcriptional expression gradients along the rostro-caudal axis emerge and whether differential NT3 levels in different muscles contribute to their establishment. In addition, our inability to generate antibodies to detect Gabrg1 protein in the spinal cord prevented us from assessing whether only subsets of vGlut1^on^ proprioceptive terminals accumulate Gabrg1 protein centrally, as would be predicted from our *in situ* hybridization experiments. GABAA receptors of different subunit composition are known to exhibit channel properties of different dynamics or magnitude [36], [55] and alterations in subunit composition might therefore, in principle, influence the effect of presynaptic inhibition at central proprioceptive synapses. A possible function of *Gabrg1* and other receptor component subunits in regulation of presynaptic inhibition will therefore be an important avenue to pursue in the future to understand the function of presynaptic inhibition in processing of sensory and motor output information.

Our work raises the interesting possibility that postsynaptic components of this presynaptic inhibitory circuit may not be expressed ubiquitously by all sensory neurons, and that NT3 signaling may influence the functionality of this pathway. Our experiments on voluntary running in the adult, although not establishing a direct link to NT3 signaling, lend support to the idea that plasticity of sensory neuron gene expression in order to dynamically adjust the needs of the circuit is a life-long process. In this context, it is interesting to mention that application of the neurotrophic factor Artemin after dorsal root injury in the adult leads to central sensory axon growth and functional restoration [56]. Moreover, viral expression of NT3 in the brainstem upon cervical spinal cord injury promotes dose-dependent axonal targeting to these areas [57], and application of NT3 to axotomized peripheral nerves promotes functionality of spinal sensory collaterals [58]. Taken together with our findings, it is likely that these interventions exhibit their potent functions at least partially through transcriptional regulation of sensory neuron gene expression. The fact that genes developmentally regulated by target-derived signals reappear in the behavioral paradigm carried out in our study in the adult suggests that expression of at least subsets of genes remains plastic also in the adult, a feature that may be important for the design of strategies interfering with pain or neuronal injury.

## Supporting Information

Figure S1 **Regulation of** ***Gabrg1*** **expression in** ***mlc^NT3^*** **mice.** *In situ* hybridization experiment demonstrating upregulation of *Gabrg1* expression in p0 L5 DRG of *mlc^NT3^* mice (right) in comparison to wild-type (left). Quantification revealed an increase in *Gabrg1^on^* DRG neurons in *mlc^NT3^* mice (L1: 2.58 fold (±0.41 SEM); L5: 2.65 fold (±0.39 SEM)) compared to wild-type (n = 3 mice each condition), but *mlc^NT3^* mice also exhibit an overall increase in proprioceptor DRG neuron numbers [21]. Although *in situ* hybridization cannot accurately quantify expression levels, side-by-side comparison of sections also revealed an apparent increase in signal intensity in DRG neurons in *mlc^NT3^* mice.
(TIF)

Table S1 (XLSX)
