# Supplementary figures and images for: Scaling Proprioceptor Gene Transcription by Retrograde NT3 Signaling
Source: PLoS One. 2012 Sep 19;7(9):e45551. doi: 10.1371/journal.pone.0045551 (PMC3447004; doi:10.1371/journal.pone.0045551)

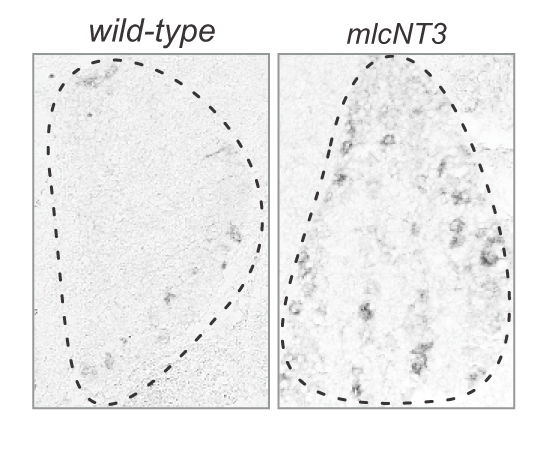

Supplement: Figure S1 — Regulation of Gabrg1 expression in mlcNT3 mice. In situ hybridization experiment demonstrating upregulation of Gabrg1 expression in p0 L5 DRG of mlcNT3 mice (right) in comparison to wild-type (left). Quantification revealed an increase in Gabrg1on DRG neurons in mlcNT3 mice (L1: 2.58 fold (±0.41 SEM); L5: 2.65 fold (±0.39 SEM)) compared to wild-type (n = 3 mice each condition), but mlcNT3 mice also exhibit an overall increase in proprioceptor DRG neuron numbers [21]. Although in situ hybridization cannot accurately quantify expression levels, side-by-side comparison of sections also revealed an apparent increase in signal intensity in DRG neurons in mlcNT3 mice. (TIF) [file pone.0045551.s001.tif]
